# Conservation at a slow pace: terrestrial gastropods facing fast-changing climate

**DOI:** 10.1093/conphys/cox007

**Published:** 2017-03-18

**Authors:** Annegret Nicolai, Armelle Ansart

**Affiliations:** 1 UMR CNRS 6553 EcoBio/OSUR, Station Biologique Paimpont, Université Rennes 1, 35380 Paimpont, France; 2 UMR CNRS 6553 EcoBio/OSUR, Université Rennes 1, 35042 Rennes cedex, France

**Keywords:** Cold tolerance, dispersal, drought resistance, extreme events, habitat alteration, species at risk

## Abstract

The climate is changing rapidly, and terrestrial ectotherms are expected to be particularly vulnerable to changes in temperature and water regime, but also to an increase in extreme weather events in temperate regions. Physiological responses of terrestrial gastropods to climate change are poorly studied. This is surprising, because they are of biodiversity significance among litter-dwelling species, playing important roles in ecosystem function, with numerous species being listed as endangered and requiring efficient conservation management. Through a summary of our ecophysiological work on snail and slug species, we gained some insights into physiological and behavioural responses to climate change that we can organize into the following four threat categories. (i) Winter temperature and snow cover. Terrestrial gastropods use different strategies to survive sub-zero temperatures in buffered refuges, such as the litter or the soil. Absence of the insulating snow cover exposes species to high variability in temperature. The extent of specific cold tolerance might influence the potential of local extinction, but also of invasion. (ii) Drought and high temperature. Physiological responses involve high-cost processes that protect against heat and dehydration. Some species decrease activity periods, thereby reducing foraging and reproduction time. Related costs and physiological limits are expected to increase mortality. (iii) Extreme events. Although some terrestrial gastropod communities can have a good resilience to fire, storms and flooding, an increase in the frequency of those events might lead to community impoverishment. (iv) Habitat loss and fragmentation. Given that terrestrial gastropods are poorly mobile, landscape alteration generally results in an increased risk of local extinction, but responses are highly variable between species, requiring studies at the population level. There is a great need for studies involving non-invasive methods on the plasticity of physiological and behavioural responses and the ability for local adaptation, considering the spatiotemporally heterogeneous climatic landscape, to allow efficient management of ecosystems and conservation of biodiversity.

## Teaser

Climate change, potentially increased by anthropogenic pressure, is still poorly considered in species status assessments for conservation. Terrestrial gastropods are expected to be greatly impacted, with subsequent effects on ecosystem functioning. We show the need to integrate physiological and behavioural traits to improve projection of the effects of climate change and conservation management for species at risk.

## Introduction

Climate is one of the most important drivers of species’ distribution and abundance. Over the last decades, the earth's climate has irrefutably warmed. For the coming century, global mean surface temperature is projected to increase by ~1°C, for the most optimistic scenario, to ~3.7°C, for the least optimistic one. The global precipitation regime is expected to change, increasing the contrast between seasons and between wet and dry regions (Meehl *et al*., 2007). In temperate regions, snow cover and snow events will decrease, and extreme events, such as heat waves and drought, heavy precipitation and storms, are expected to occur more often (Stocker *et al*., 2013). Rapid variation of climate is expected to alter species’ life-history traits (for example, phenology, Fabina *et al*., 2010) and biotic interactions (e.g. Wernegreen, 2012), which might lead to range contractions or even extinctions (Thomas *et al*., 2004, 2006).

**Figure 1:**
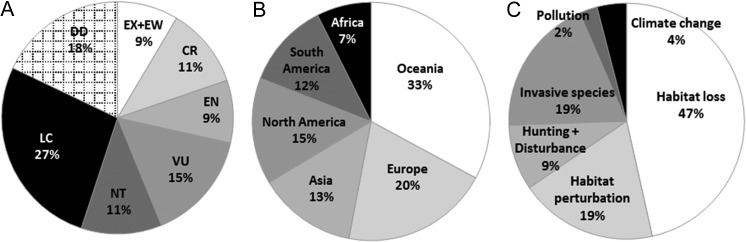
Terrestrial gastropods on the IUCN Red List of Threatened Species™ (IUCN, 2016). (**A**) Global distribution of assessed terrestrial gastropods (*n* = 2070) in the red list categories Abbreviations: CR, critically endangered; DD, data deficient; EN, endangered; EX+EW, extinct and extinct in the wild; LC, least concern; NT, near threatened; and VU, vulnerable. (**B**) Geographical origin of terrestrial gastropods in the CR category (*n* = 283). (**C**) Threats (*n* = 2153) encountered in all red list categories for assessed terrestrial gastropods (*n* = 2070). One species can face more than one threat.

Terrestrial ectothermic organisms may be particularly affected by modifications of local climate conditions, because body temperature and, subsequently, basic physiological functions, depend on environmental temperature (Gillooly *et al*., 2001; Deutsch *et al*., 2008). Among ectotherms, terrestrial gastropods (i.e. land snails and slugs) are particularly prone to climate change for the following reasons. First, their activity and physiology are highly sensitive to local temperatures (Cameron, 1970; Bailey, 1975), and many species enter a state of dormancy (aestivation and/or hibernation) when conditions are unfavourable for activity (e.g. Heller and Ittiel, 1990; Iglesias *et al*., 1996). Second, their moist skin and the secretion of a mucus trail for locomotion make them particularly sensitive to low hygrometric conditions (e.g. Young and Port, 1989). Third, moreover, their proverbial slowness and high cost of movement strongly limit their ability to escape a hostile environment actively (Denny, 1980). Although widely used as witnesses of previous climate changes (Goodfriend, 1992), terrestrial gastropods have so far received little attention regarding the potential impact of current rapid climate change on their distribution, compared with other taxa. This is surprising for the following reasons. First, with ~24 000 described species, inhabiting a large range of habitats, terrestrial gastropods are one of the most diverse group of land animals (Lydeard *et al*., 2004), playing a primordial role in ecosystem functioning specifically by aiding in decomposition, nutrient cycling and soil-building processes (Mason, 1970a, b; Jennings and Barkham, 1979; Prather *et al*., 2013), providing food and essential nutrients to wildlife (South, 1980; Churchfield, 1984; Frest and Johannes, 1995; Martin, 2000; Nyffeler and Symondson, 2001), and determining plant community structure (Hulme, 1996; Peters, 2007). Second, 1105 species worldwide appear on the IUCN red list as extinct, critically endangered, endangered or vulnerable (www.iucnredlist.org), and many more are listed regionally and nationally (Fig. 1A). Third, many species are serious pests that lead to crop damage and pesticide spreading (Barker, 2002) as well as negative impacts on natural habitats and native biodiversity (Cowie, 2011). Fourth, some gastropods are pathogen vectors (Rowley *et al*., 1987; Graeff-Teixeira, 2007).

Models of future distribution of gastropod species tend to predict range shifts and an increase of diversity with latitude and altitude. These models are based on climate projections that predict higher temperatures and more precipitation at high latitudes and altitudes compared with low latitudes and altitudes, where climate is expected to become drier (Willis *et al*., 2006; Müller *et al*., 2009; Hof, 2011; Beltramino *et al*., 2015). Although such models have a general interest, their reliability is limited. For example, most of the critically endangered species live on islands (Oceania) or in Europe and North America, where the climate change will largely interact with anthropogenic pressures (Fig. 1B and C; Gibson *et al*., 2009). In fact, it has been demonstrated that climate change could push gastropod species to the brink of extinction, e.g. *Rhachistia aldabrae* was believed to be extinct (Gerlach, 2007), but a few specimens were rediscovered in 2014 (Amla, 2014). Modifications in species’ distribution and abundance without a northward shift of the range were also reported (Baur and Baur, 1993, 2013; Peltanova *et al*., 2012; Pearce and Paustian, 2013).

**Figure 2:**
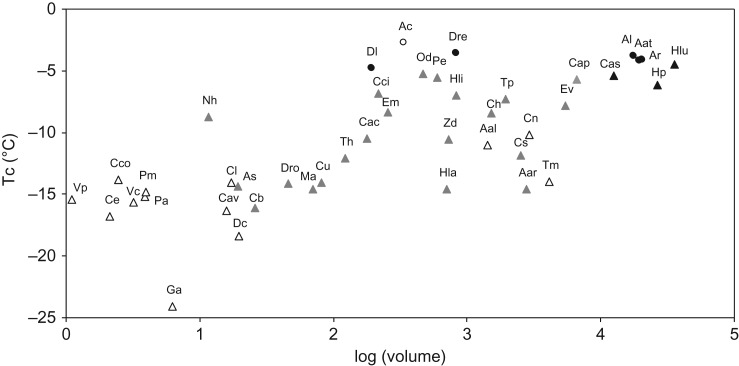
Relationships between the temperature of crystallization (Tc) and the volume of 43 species of slugs and snails. Triangles indicate shelled species and circles slugs. White symbols indicate freeze-intolerant species, black symbols partly freeze-tolerant or freeze-tolerant species and grey symbols indicate species with unknown cold-tolerance strategy. Data are from Ansart *et al*. (2014) and personal observations A.A. except for species Dl and Ac (Storey *et al*., 2007), Dre (Cook, 2004), Al, Aat and Ar (Slotsbo *et al*., 2012), Aal (Riddle, 1981), Dc and Ga (Riddle and Miller, 1988), Cav (Koštál *et al*., 2013), Vp (Schmid, 1988) and Aar (Stöver, 1973), Tm (Franke, 1985). Sample size ranges from five to 110 individuals depending on species. Only Tc values obtained during the cold season have been considered. Volume was estimated as the mean between the volume of a cone and that of an ellipsoid for shelled snails (see Ansart *et al*., 2014) and as the volume of a cylinder for slugs. Mean size estimations were extracted from Welter-Schultes (2012), Kerney and Cameron (1999) and Rowson *et al*. (2014) for European species and from Pilsbry (1940, 1948) for American species. Abbreviations: Aal, *Anguispira alternata*; Aar, *Arianta arbustorum*; Aat, *Arion ater*; Ac, *Arion circumscriptus*; Al, *Arion* ‘*lusitanicus*’ *= vulgaris* (invader); Ar, *Arion rufus*; As, *Abida secale*; Cac, *Cochlicella acuta*; Cap, *Cantareus apertus*; Cas, *Cornu aspersum*; Cav, *Chondrina avenacea*; Cb, *Clausilia bidentata*; Cci, *Ciliella ciliata*; Cco, *Columella columella*; Ce, *Columella edentula*; Ch, *Cepaea hortensis*; Cl, *Cochlicopa lubrica*; Cn, *Cepaea nemoralis*; Cs, *Cepaea sylvatica*; Cu, *Candidula unifasciata*; Dc, *Discus cronkhitei*; Dl, *Deroceras laeve*; Dre, *Deroceras reticulatum*; Dro, *Discus rotundatus*; Em, *Ena montana*; Ev, *Eobania vermiculata*; Ga, *Gastrocopta armifera*; Hla, *Helicigona lapicida*; Hli, *Hygromia limbata*; Hlu, *Helix lucorum*; Hp, *Helix pomatia*; Ma, *Macrogastra attenuata*; Nh, *Nesovitrea hammonis*; Od, *Oxychilus draparnaudi*; Pa, *Pupilla alpicola*; Pe, *Pomatias elegans*; Pm, *Pupilla muscorum*; Th, *Trochulus hispidus*; Tp, *Theba pisana*; Vc, *Vallonia costata*; Vp, *Vallonia perspectiva*; Wm, *Triodopsis* (*Webbhelix*) *multilineata*; and Zd, *Zebrina detrita*.

Conservation management plans aim to protect ecosystems and biodiversity, but they often lack approaches that take the ecological impacts of climate change into account, as well as specific organism responses and interactions. Furthermore, conservation strategies typically target the ecological integrity of large areas, a scale inadequate for taxa such as terrestrial gastropods, which are constrained by low mobility or thermal barriers within the habitat. The lack of knowledge on organisms’ responses to climate change and on how effects of extreme weather events can be mitigated by adequate habitat management is striking. There is a crucial need to incorporate physiological and behavioural specific traits into projection of ecological effects of climate change (Deutsch *et al*., 2008) when conceiving management or restoration plans of protected areas (Evans *et al*., 2015). For example, the Committee of Status of Endangered Wildlife in Canada (COSEWIC) identified droughts and absence of snow (Augspurger, 2013) as ongoing threats in all of the species at-risk assessments of terrestrial gastropods (14 species; see www.sararegestry.gc.ca for species status reports). However, none of them could clearly identify the scope and severity of climate change impact because specific physiological and behavioural knowledge is lacking. Only partial knowledge on thermal tolerance of model organisms (e.g. Staikou, 1999; see also Table 1 and Fig. 2) is available.

**Table 1:** Comparison of cold tolerance strategies in three species of European Helicidae

Parameter	*Helix pomatia*	*Cornu aspersum*	*Cepaea nemoralis*
Annual cycle	Hibernation: 5–6 months Aestivation: few days	Hibernation/aestivation: highly variable across Europe (6–0 months)	Hibernation: 5–6 months Aestivation: few days
Shell breadth	30–50 mm	25–40 mm	18–25 mm
Cold-tolerance processes	Tc = −2°C (activity) Tc = −6°C (dormancy) LT_50_ = −10°C (2 h exposure)	Tc = −3°C (activity) Tc = −5°C (dormancy) LT_50_ = −10°C (2 h exposure)	Tc = −4°C (activity) LT_50_ = −10°C (2 h exposure in activity) Tc = LLT = −10°C (dormancy)
Cold-tolerance strategy	Partly freeze tolerant	Partly freeze tolerant	Partly freeze tolerant (activity)Freeze avoidant (dormancy)
Distribution	South-Eastern Europe to England and Scandinavia	North Africa to North Western Europe (The Netherlands, England) and eastwards to the Rhine Valley	Central Europe from Mediterranean to England and eastwards to Poland
Status	Protected in Europe (Appendix III, Bern Convention)	Invasive in North and South America, Australia, South Africa and New Zealand	Invasive in North America
References	Nietzke (1970); Lind (1988); Kerney and Cameron (1999); Nicolai *et al*. (2005, 2011, 2012a, 2015); Ansart *et al*. (2014); personal observation A.N.	Kerney and Cameron (1999); Ansart *et al*. (2001, 2002, 2010, 2014); Nicolai *et al*. (2005); personal observations A.A. and A.N.	Kerney and Cameron (1999); Ansart *et al*. (2014); personal observations A.A. and A.N.

Abbreviations: LT_50_, lethal temperature for 50% of individuals; LLT, absolute lower lethal temperature; Tc, temperature of crystallization.

Currently, the threats of climate change are documented in only 87 of 283 critically endangered terrestrial gastropods worldwide (Fig. 1C; IUCN, 2016). Although habitat loss and perturbation, invasive species and pollution may increase the impact of climate change, in many of the remaining species the impact of the threats of climate change are unknown. In the threat calculation for a species at-risk assessment, ‘climate change’ is divided into different threat categories illustrating effects on species (for complete information on these categories, see Salafsky *et al*., 2008 and http://cmp-openstandards.org/using-os/tools/classification-beta-v-2-0). These categories are not always evident to differentiate. For example, ‘changes in temperature regimes’ (threat 11.3) and ‘changes in precipitation and hydrological regimes’ (threat 11.4) are, in general, closely linked. Here, we gathered some aspects of terrestrial gastropod physiology and species’ strategies, including our own research work, which may provide a perspective for increasing the understanding of potential impacts of climate change and a gaining integration of species traits in conservation management. We address the following four main climate change threats, critical for terrestrial gastropods: (i) winter temperature and snow cover; (ii) drought and high temperature; (iii) extreme events; and (iv) habitat loss and fragmentation.

## Winter temperature and snow cover

Climate warming includes changes of mean temperature with the effect of delaying the cold season, but also increasing the variability of temperature in the litter and upper soil layers as a result of the absence of snow, especially in autumn and spring when temperatures fluctuate around 0°C (Augspurger, 2013). Delaying the winter season might have an impact on phenology of terrestrial gastropods. Winter dormancy is triggered by photoperiod and temperature (Bailey, 1981; Jeppesen, 1977), and reproduction is possible at suitable temperatures with decreasing photoperiod (Nicolai *et al*., 2010). Terrestrial gastropods that change phenology by later entry into dormancy and/or autumn reproduction are susceptible to be exposed to below-zero temperatures and/or have reduced energy reserves for winter survival, respectively. Given that winter survival increases with size in many terrestrial gastropod species (in *Cepaea nemoralis*, Oosterhoff, 1977; in *Arianta arbustorum*, Terhivuo, 1978; Baur and Baur, 1991), juveniles from autumn reproduction might be particularly vulnerable, especially if they do not reach the crucial shell size below which they are unable to hibernate (Biannic and Daguzan, 1993). The length of dormancy is also important for genital tract maturation and gamete genesis, assuring the success of future reproduction (Bonnefoy-Claudet and Deray, 1984; Gomot and Gomot, 1991). When dormancy of *C. nemoralis* was reduced from 5–6 to 3 months, no spring reproduction occurred, and the albumen gland had increased by only 36% of its initial dry mass compared with 91% increase after 5 months (unpublished data A.N. for Annegret Nicolai). Although some species have a highly variable length of dormancy depending on the geographical origin of the population, other species need a long dormancy period, such as the Roman snail *Helix pomatia* (Lind, 1988) dormancy period. For example, hibernation in *C. aspersum* lasts >7 months in Scotland (Crook, 1980), 6 months in Wales (Bailey, 1981), ~5 months in North-Western France (Lorvelec and Daguzan, 1990) and 4 months in North-Western Spain (Iglesias *et al*., 1996), whereas some Mediterranean populations do not hibernate (Madec, 1989). Changing phenology has consequences for population dynamics because of the possibility of higher winter mortality, low juvenile winter survival from autumn reproduction and reduced spring reproduction.

Survival to sub-zero temperatures is partly related to behaviour, such as the ability to occupy buffered microsites (Heller and Dolev, 1994; Nicolai *et al*., 2011) and the orientation of the apex in the hibernation position (Carney, 1966; Terhivuo, 1978; Baur and Baur 1991). At a physiological level, winter mortality can be caused by insufficient energy reserves to endure long periods of inactivity (Storey and Storey, 1990, 2004; Bailey and Lazaridou-Dimitriadou, 1991; Heller and Dolev, 1994; Pakay *et al*., 2002) and by a low ability to tolerate the exposure to sub-zero temperatures. Cold hardiness strategy is thus crucial for the maintenance and development of a species in a cold-constrained habitat (Bale and Walters, 2001; Pither, 2003; Bale and Hayward, 2010; Chown *et al*., 2010). There are currently two main categories of physiological responses to sub-zero temperatures, freeze avoidance and freeze tolerance, although many intermediate response strategies are possible. Freeze-tolerant organisms generally have a poor ability to supercool (i.e. the ability to maintain body fluids at a liquid state below the freezing point), between −5 and −10°C, allowing slow freezing of tissues and thus sufficient time to implement protection mechanisms. In some snail species, ice nucleating agents decrease the supercooling ability (e.g. gut bacteria; Ansart *et al*., 2010; Nicolai *et al*., 2015). On the contrary, in freeze-avoidant organisms, for which ice formation in tissues is lethal, the supercooling ability will be enhanced, often in association with the synthesis of large amount of antifreeze substances (for more information on these strategies, see, for example, Ramløv, 2000; Zachariassen and Kristiansen, 2000; Block, 2003).

Winter mortality as a result extreme cold temperatures appears to be a key factor in shaping the distribution and abundance of land gastropods (Pfenninger, 2004; Horsák *et al*., 2013; Horsák and Chytry, 2014). Terrestrial gastropods are largely present in regions with harsh cold seasons; in northwest Europe, 35 species are recorded above the Arctic Circle and 44 species can be found at altitudes higher than 2000 m (Kerney *et al*., 1983; Kerney and Cameron, 1999), and in Canada, ~30 species live at high altitudes in the Rocky Mountains and in arctic provinces (Grimm *et al*., 2009; Forsyth RG pers. communication).

Cold tolerance strategies are still poorly known within the group of terrestrial gastropods, but all strategies from freezing avoidance to tolerance can be encountered. Some species have a short generation time, and only eggs will persist during winter (e.g. the slug *Lehmannia marginata*; Cook and Radford, 1988). For all studied species, eggs are killed by ice formation but can supercool more or less depending on the species [temperature of crystallization (Tc) = −3.9 to −14.7°C in 34 different species; Tc is the temperature at which body fluids start to freeze; unpublished data A.A. and A.N. for the two authors). In species for which adults hibernate, the comparison of 31 terrestrial European snails from 13 different families revealed a strong phylogenetic constraint on the Tc, highly dependent on both water mass and water content (see Fig. 2; Ansart *et al*., 2014). The cold hardiness strategy has been extensively studied for only few species; all small species with high supercooling ability are freezing avoidant, in contrast to large snail species, which can supercool poorly but tolerate freezing of body fluids to some extent (from a few hours to a few days). All slug species, particularly sensitive to inoculative freezing, appear to be freezing tolerant (Fig. 2). Even if knowledge is still too scarce to conclude undoubtedly, only the smallest species seem able to survive extreme cold temperatures during winter. This hypothesis is supported by several studies offering evidence that land snail communities are less diverse at high altitudes and latitudes, where small taxa become more frequent (Hausdorf and Hennig, 2003; Nekola *et al*., 2013; Baur *et al*., 2014; Horsák and Chytry, 2014; Schmera and Baur, 2014; Forsyth and Oldham, 2016). With climate warming, we would expect the gastropod community to become richer in bigger species, with consequences for competition, trophic webs, vegetation grazing and soil dynamics.

Under global warming, climatologists predict a decrease of snow events and a thinning of snow cover. Snow cover plays a very important buffer role. When present, soil temperature is perfectly constant near 0°C, i.e. above the freezing point of the fluids of living organisms. If absent, high variation of temperature can be a real threat for species survival and considerably modify future distribution predictions. In *Chondrina clienta* and *Balea perverse*, two sympatric species with different cold tolerance capacities, survival was significantly higher in snow-covered microsites compared with exposed ones, at 90 vs. 79% and 100 vs. 88%, respectively (Baur and Baur, 1991). In recent years, we have compared cold tolerance in three Helicidae species: the large edible snails *Cornu aspersum* (brown garden snail) and *Helix pomatia* (Roman Snail), and the smaller *C. nemoralis* (grove snail), revealing that closely related species within the same family can have very different responses to climate change (Table 1). *Cornu aspersum* and *C. nemoralis* are invasive in some parts of the world, owing in particular to the development of human-related traffic. In some places, they are considered to be an agricultural pest (e.g. in USA and Canada, Ansart *et al*., 2009; Cowie *et al*., 2009). On the contrary, *H. pomatia* was common in many parts of Europe until the end of the 20th century (Nietzke, 1970), but habitat loss through agriculture and urbanization diminished important populations in some parts of Europe, such as Germany. As consequence, the species has been protected in Europe since 2002 (Appendix III of the Bern Convention). We found that the three species are partly freezing tolerant in activity, because they can bear body freezing (but only for a very short time (Table 1)). During the winter, while *H. pomatia*, partly freezing tolerant, relies on permanent snow cover for winter survival, *C. nemoralis*, freeze-avoidant with a higher supercooling capacity, can survive longer periods of frost. *Cepaea nemoralis* would then be less vulnerable than *H. pomatia* to high variations in winter temperature, which are expected to occur more frequently at high latitudes when snow cover is absent (Augspurger, 2013). Different geographical populations show slight variations in cold hardiness processes, but an adaptive and/or plastic response to local climate is difficult to prove because differences in individual size and water compartment interfered with environmental conditions (Ansart and Vernon, 2004; Nicolai *et al*., 2005, 2012a; Gaitan-Espitia *et al*., 2013). The loss of snow cover is the major threat of climate change in temperate regions, because it directly affects winter survival and indirectly affects reproductive success in some bigger terrestrial gastropods (data for smaller gastropod species are lacking).

## Drought and high temperature

Temperature has a fundamental impact on physiological processes that determines the performance of ectotherms relative to temperature (Angilletta, 2009). Thermal performance curves (Gillooly *et al*., 2001), which describe this relationship, are asymmetric for most ectotherms, and upward shifts of global temperature bring organisms much closer to critical limits, leading to the risk of local extinction, especially for species that cannot rapidly shift their distribution (Dillon *et al*., 2010), such as terrestrial gastropods.

As a consequence of their soft and permeable integument, air breathing and mode of locomotion, land gastropods are particularly sensitive to desiccation (Machin, 1964). Body temperature regulation is mainly achieved by the active research of a buffered microhabitat and metabolic down-regulation (daily torpor or aestivation). Although many scientists have been interested in the upper thermal limit and adaptations to heat of intertidal species, which endure daily highly variable ecological conditions (e.g. Marshall and McQuaid, 2011), far fewer have studied precisely heat resistance and the underlining mechanisms in land snails and slugs. At the community level, whereas the surface-to-volume ratio of globular shells would be more beneficial for facing dehydration in high-temperature conditions (Ansart *et al*., 2014), oblong and thick shells would prevent desiccation and facilitate access to sheltered microhabitats (Giokas *et al*., 2005). Lacking a shell, slug species are supposed to be much more sensitive to heat stress (Thompson *et al*., 2006).

Usually, snails can cope with drought periods in the summer. Although *H. pomatia* aestivates for a short period, *C. aspersum* can endure several months in the Mediterranean region (Table 1), and snails of semiarid to arid regions are inactive for the whole year except for a few rainy days (e.g. 6–12 days/year in *Cristataria genezarethana*; Heller and Dolev, 1994). Mortality in snails from semiarid to arid regions is usually low; 5% in *Cristataria genezarethana* (Heller and Dolev, 1994) and 14% in *Rhagada convicta* (Johnson and Black, 1991), whereas *H. pomatia* in a continental climate has a mortality rate that varies between age classes from 6 to 20% (Starodubtseva and Dedkov, 2003). However, during a drought of 1 month immediately after arousal from hibernation when snails had to recover from winter fasting, mortality was up to 70% in *H. pomatia* in Germany (Nicolai *et al*., 2011). Richardson (1974) observed direct lethal effect of high temperatures in a sand-dune population of *C. nemoralis*. For the same species, Chang and Emlen (1993) showed that repartition of the individuals in the landscape was strongly constrained by high temperatures and low humidity. Linked to urban development, subsequent increase in the soil temperature near Basel (Switzerland) might have triggered the extinction of several populations of the land snail *Arianta arbustorum*, mainly explained by the limited heat resistance of eggs (Baur and Baur, 1993). In the same species, it has also been demonstrated that the sperm length was reduced at higher temperatures, thereby affecting reproductive success (Minoretti *et al*., 2013). In the African species *Achatina fulica* and *Macrochlamys indica*, a longer aestivation was related to a weaker viability of allosperm stored in the spermatheca, a longer gestation period because of the time necessary to meet energy needs, and a reduced number of eggs per brood (Raut and Ghose, 1982).

For species inhabiting arid regions, being active for very short periods and requiring several years to reach maturity (e.g. Yom-Tov, 1971; Heller and Dolev, 1994; Arad *et al*., 1995), longer droughts can have important consequences on population dynamics, as for the endangered Corsica snail *Tyrrhenaria ceratina*, for which activity periods are extremely short in spring and autumn depending on humidity (Charrier *et al*., 2013).

Heat tolerance is diverse among terrestrial gastropods (e.g. Riddle, 1990). Given that most species spend drought periods hidden in a buffered shelter, with their shell aperture closed with an epiphragm, costs associated with aestivation are also the production of chemical compounds that allow for maintenance of cell function. The Mediterranean species *Xeropicta derbentina* is particularly well adapted to high temperatures, with individuals enduring hot hours of the day agglutinated on the top of vegetation. In this species, calcium cells playing a role in osmoregulation (Dittbrenner *et al*., 2009; Scheil *et al*., 2011), heat shock proteins and antioxidant defences (Troschinski *et al*., 2014) have been shown to be implicated in heat tolerance. In the less heat-adapted species *H. pomatia*, we observed via a metabolomic fingerprinting method an increase of polyols, such as myo-inositol and glycerol, and sugars, such as maltose, that might protect the cell against dehydration (Nicolai *et al*., 2011). Moreover, cholesterol was found in high concentration, which is responsible for the maintenance of membrane fluidity at high temperatures (Robertson and Hazel, 1997). The high concentration of succinid acid indicated the use of an anaerobic mollusc-specific glycolytic pathway that produces more energy than fermentation (Livingstone, 1991) also observed in some slugs during freezing (Storey *et al*., 2007). These compounds implied a stress reaction to a prolonged drought, with an unusual timing involving high costs.

We can also expect different selective intra-specific effects of climatic pressures on land gastropods. Individuals with light-coloured shells have been suggested to tolerate solar radiation and heat better (e.g. *C. nemoralis*, Richardson, 1974; *C. aspersum*, Lecompte and Madec, 1998; *Cepaea vindobonensis*, Staikou, 1999; *Theba pisana*, Johnson, 2011). In *C. nemoralis*, for which shell polymorphism has been much studied, the higher frequency of light shells in open habitats compared with dark ones (Ożgo, 2005) has been related to differences in mortality (Tilling, 1983), activity (Chang, 1991) and behavioural thermoregulation (Jones, 1982) in relationship with the opioid system (Kavaliers, 1992).

## Extreme events

### Flood

If floods are a normal and important element of biodiversity maintenance in certain ecosystems (Ilg *et al*., 2009), they can also directly or indirectly threaten terrestrial soil faunas, for several reasons, as follows: (i) air breathing becomes impossible; (ii) water intrudes into body compartments (swelling); (iii) individuals can uncontrollably be displaced and/or (iv) be contaminated by toxic substances from the water; and (v) environmental conditions can be impacted by flooding (Plum, 2005). Abundances and biomass of soil invertebrates are immediately reduced by flooding. The effect is generally reversible and is normally compensated during the next soil dry period, but flooding events that are too frequent can prevent communities from recovering and affect the environmental conditions more harshly (Plum, 2005).

There have been few studies concerned with the effects of flooding on land gastropod communities or species. Along a gradient of flood frequency in Danubian floodplain forests, Čejka *et al*. (2008) found that land snail communities were less rich, with lower abundance, when floods were frequent, compared with other sites. When confronted by flooding, slugs and snails actively migrate, but their ability to move is insufficient to escape brutal inundation. They can be transported passively, thereby colonizing new habitats (by water itself, driftwood, or large animals; Dahl *et al*., 1993; Ożgo *et al*., 2015). They can avoid drowning by climbing onto emerging substrates or floating vegetation or by using air-filled cavities in the soil (Dahl *et al*., 1993).

Flood impacts on land gastropods can be difficult to generalize. Some terrestrial gastropods, such as *C. nemoralis*, might be able to survive sudden hypoxic conditions for several hours. Active adults of *C. nemoralis* were exposed to N_2_ for 15 h and tested for long-term survival; all snails survived and reproduced (unpublished data A.N.). Antioxidant defences have been shown to be highly implicated in tolerance to hypoxia/anoxia occurring during dormancy periods in *C. aspersum* (Welker *et al*., 2016) and *H. pomatia* (Nowakowska *et al*., 2015). Given that the majority of gastropod species spend some time with low metabolism, we could expect such a tolerance to be highly shared among the taxon.

In the North American snail *Anguispira kochi* the main threat for the Canadian range is increased risk of flooding of the habitat (COSEWIC, in press). The water level of Lake Erie has been high for a few years owing to higher precipitation, which increases the impact of storms, completely immersing smaller islands and eroding the habitat. Population maintenance in stochastically flooded occurrence sites might depend on the population density, dispersal capacity in the fragmented landscape and the spatiotemporal pattern of the occurrences of storms and flooding, but also on hypoxia tolerance.

### Storms and hurricanes

Species respond in different ways to large-scale habitat modifications attributable to storms and hurricanes with respect to population density, absolute spatial variability or relative spatial variability (Bloch and Willig, 2006). Storms can change the pattern of spatial organization of the gastropod community through cross-scale interactions between local species demographics and human-shaped landscape configuration of patches (Willig *et al*., 2007). In tropical forests, gaps in the canopy triggered by hurricanes are thought to enhance mortality of gastropods by desiccation stress, mostly affecting eggs and young stages. At the same time, the deposition of organic matter on the soil provides supplementary food resources and humid microhabitat. In two endangered Hawaiian tree species, *Achatinella mustelina* and *Achatinella sowerbyana*, between-tree movements might be mostly passive, occurring during violent wind storms (Hall and Hadfield, 2009). This results in heterogeneous responses of species, with some being favoured by such events in contrast to others, and some exhibiting variable trends (Bloch and Willig, 2006).

In the case of the endangered species *T. ceratina*, climate change increased wind speed and changed wind direction at the locality, thereby allowing storms that erode the coastal habitat at an increasing frequency (Charrier *et al*., 2013). The highest abundance of the species was measured on the marine terrace ridge immediately behind the beach, probably related to the vegetation community providing food and shelter. Parts of the ridge are eroded by storms, thereby affecting the population size. Retreat to lower parts of the sand savannah behind the dunes, which are usually not affected by storms, seems to be negated by the presence of a lower gravel-soil layer in the sand. This layer originates from the construction of the urban zone bordering the habitat and interferes with deep burrowing behaviour (up to 50 cm deep) related to dormancy periods.

### Fire

Fire can result from long droughts that might increase in frequency and duration, especially in Mediterranean to continental and temperate climate. Fire can directly affect the survival of terrestrial gastropods (Nekola, 2002; Bros *et al*., 2011) or indirectly by reducing the wood, litter and mulch layer on the soil surface (Bellido, 1987). Fire reduces and modifies organic substrates and residues that are sources of nutriments, buffering and sheltering; it changes microclimate, such as heating of bare soil and increasing soil evaporation (reviewed by Saestedt and Ramundo, 1990; Knapp *et al*., 2009). In the tall grass prairie in Manitoba, the gastropod community was impoverished a few years after fire; unburned prairie hosted more aquatic and terrestrial species than burned prairie (unpublished data A.N.). In Mediterranean regions, Santos *et al*. (2009) showed that 4 years after the perturbation, the gastropod community was dominated by xerophilous species, whereas forest species dominated in unburned sites. Recolonization of burnt patches by terrestrial gastropods depends on their survival in microsites within the habitat (Kiss and Magnin, 2003, 2006). Severely burnt patches display a higher pH, possibly attractive to gastropods (Hylander, 2011). Post-fire management can increase post-fire survival by restoring the humus–litter layer and providing moist microhabitat (Bros *et al*., 2011). Even if resilience of gastropod communities to fire events is high, a long time is required to return to the initial state (Kiss and Magnin, 2006), whatever the post-fire management type (see Bros *et al*., 2011). It is expected that an increased frequency of fire and a loss of microsites as refugia within the habitat, triggered by the combined effect of climate change and human impact, will weaken their sustainability and consequently affect litter decomposition and nutrient cycling.

## Habitat loss and fragmentation

Interacting with human-driven changes in land use, climate change is expected to affect landscape structure (Opdam and Wascher, 2004; Vos *et al*., 2008). Habitat loss and habitat fragmentation are two distinct processes, even though they are often confounded in experimental and field studies, because they generally occur together (McGarigal and Cushman, 2002; Fahrig, 2003). Habitat loss generally has a negative effect on biodiversity, directly by impacting species richness, population abundance, species distribution and genetic diversity, and indirectly by affecting species interactions and population growth rate (for a review, see Fahrig, 2003).

Few examples document the effect of climate change-induced habitat loss on gastropod species, independently of fragmentation. In Lake Erie islands (North America), habitat loss results from the disruption of natural erosion–deposit cycles owing to changes in the frequency and intensity of precipitation and storms and to the human activity of sand mining. This disruption affects the habitat of the endangered species *Allogona profunda* (COSEWIC, 2014). A comparable situation is described for the Corsica snail *T. ceratina* (see section ‘*Storms and hurricanes*’; Charrier *et al*., 2013).

Habitat loss can drive species to occupy suboptimal patches, leading to reduced fitness. For example, a change in resource availability, and thereby energy and types of nutrients provided, can impact reproduction and egg quality in *C. aspersum* (Nicolai *et al*., 2012b), such as egg shell thickness, which can also lead to secondary effects on heat tolerance (Nicolai *et al*., 2013).

Models have highlighted the existence of an extinction threshold in habitat availability, below which the population size does not allow its maintenance (e.g. Flather and Bevers, 2002). Moreover, the existence of such a threshold has also been theoretically highlighted for the fixation time of selectively neutral genotypes by genetic drift (Ezard and Travis, 2006). Both thresholds are affected by habitat shape and spatial correlation (Ezard and Travis, 2006), supporting the necessity to study habitat loss and fragmentation simultaneously when considering the impact of climate change on local populations.

The consequences of fragmentation *per se* are more diverse and less clear than those of habitat loss (Robinson *et al*., 1992; Harrison and Bruna, 1999), with negative impacts involving a reduction of the population size, edge effects and reduced flow between patches, intensifying isolation, thereby decreasing genetic diversity and increasing inbreeding rate, leading eventually to the extinction of local population (Fahrig, 2003 and references therein). Positive impacts of habitat fragmentation on population maintenance have also been demonstrated, such as the persistence of inter-specific interactions (predator–prey relationship, competition); heterogeneity of patch conditions can also avoid simultaneous extinction of a whole population (Fahrig, 2003). Moreover, the matrix quality can considerably influence the impact of habitat fragmentation on biodiversity (Franklin and Lindenmayer, 2009). Taxa with low mobility, such as terrestrial gastropods, are supposed to be particularly sensitive to habitat fragmentation. Although dispersal in minute gastropods is mainly passive (wind, water, animal, mainly birds and human transport; e.g. Dörge *et al*., 1999; Aubry *et al*., 2006; Ożgo *et al*., 2015; Simonová *et al*., 2016), active dispersal is expected to play an important role at a local scale (Aubry *et al*., 2006). Active dispersal is known to be a highly variable trait, within and among populations, as a response to cost–benefit balance, depending on the species traits and environmental context and with potential consequences on population dynamics (Bonte *et al*., 2012; Clobert *et al*., 2012). This variability of active dispersal depends on climatic conditions (Hall and Hadfield, 2009; Dahirel *et al*., 2014), species (see Kramarenko, 2014 and references therein) and individual size (Baur and Baur, 1988; Honek and Martinkova, 2011), age and/or reproductive status (Tomiyama and Nakane, 1993, Dahirel *et al*., 2014) and habitat exploitation (sedentary vs. nomadic species, Murphy, 2002; Edworthy *et al*., 2012; specialist vs. generalist species, Kappes *et al*., 2009; Dahirel *et al*., 2015). As an illustration, in the minute European land snail *Punctum pygmaeum* (shell breadth approximately 1.2–1.5 mm), the mean distance travelled over a 12 h period is 47 mm (Baur and Baur, 1988), whereas in the large Australian *Hedleyella falconeri* (shell breadth approximately 90–100 mm), individuals moved 9 m on average per night (Murphy, 2002), and in the endangered *Allogona townsendiana*, displacement during 3 years was 32.2 m (Edworthy *et al*., 2012).

Fragmentation can alter gastropod dispersal cost in two ways: directly, as the cost for crossing hostile matrix is increased, and indirectly, as movements of vector species, such as mammals, can also be affected by landscape structure changes (Kappes *et al*., 2009). Moreover, alteration of the habitat and its consequences (reduced resources, higher density, microclimatic changes and community modification) can modify the dispersal behaviour of species. In general, fragmentation is negatively correlated with emigration propensity (Bonte *et al*., 2006; Schtickzelle *et al*., 2006). If a boundary-crossing avoidance behaviour has effectively been observed in gastropods (Baur and Baur, 1988; Giokas and Mylonas, 2004), in some species, such as the brown garden snail *C. aspersum*, exploration increased with higher fragmentation on an urbanization gradient (Dahirel *et al*., 2016). In the endangered Canadian species *A. townsendiana*, the individual home-range area was highly variable (from 18.4 to 404.4 m²), depending on habitat quality; in habitats where stinging nettle (food source and shelter) and coarse woody debris (reproduction sites) were present, the home range was significantly smaller (Edworthy *et al*., 2012). In a comparative analysis (20 European Helicoidea species), Dahirel *et al*. (2015) showed that several traits linked to mobility were phylogenetically constrained (e.g. locomotion speed), whereas others were independent of phylogeny (e.g. exploration propensity, path sinuosity).

Several field or experimental studies gave interesting insights into the consequences of fragmentation for gastropod species. In small conservation forests, litter mollusc species assemblages depended not only on local environmental variables but also on surrounding landscapes, with open fields having a negative impact on species richness and composition (Götmark *et al*., 2008), somehow counterbalancing the conclusion from Cameron and Pokryszko (2004); gastropod faunas can survive in very small fragments of suitable habitat. Moreover, connectivity of habitat patches has been shown to be important for maintaining a diversity of taxa with low dispersal ability, such as gastropods (Knop *et al*., 2011). In an experimentally fragmented grassland, a 3 year survey of six land snail species allowed the demonstration of a significant impact of fragmentation on all species, but with specific differences in population size reduction, extinction rate and recolonization probability (Stoll *et al*., 2009). Species with a larger body (more mobile), longer activity period and short generation time were less susceptible to fragmentation. The fragment size was negatively correlated with the extinction rate and positively correlated with the recolonization probability, highlighting the importance of edge effects, affecting both the composition of vegetation and microclimatic conditions on the edge zone (Stoll *et al*., 2009). In the same experimental system, the reduced density of herbivores (mainly gastropods) and the vegetation shifts in the fragments decreased the grazing intensity to *Trifolium repens* seedlings, thereby amplifying the modification of the habitat quality (Ledergerber *et al*., 2002). In deciduous forests, fragmentation affected more forest-specialized than euryecious and matrix species, with main effects being generated by edge effects on environmental conditions (Kappes *et al*., 2009).

Facing rapid changes of their habitat, some species have proved a high evolvability of some traits, such as *C. nemoralis*. In the British Isles and The Netherlands, large-scale habitat changes (opening and warming) triggered a rapid adaptive change in shell morph frequencies; lighter and unbanded shells were more frequent, which could be advantageous against insolation and predators compared with darker and banded shells (Ożgo and Schilthuizen, 2012; Cameron and Cook, 2013; Schilthuizen, 2013). Responses to habitat loss and fragmentation appear then as an idiosyncratic trait, highlighting the necessity to conduct studies at the population level. Fast adaptation might confer some ability to maintain a population in unstable and rapidly changing habitat for some species (Ożgo, 2011), but it remains difficult, if not impossible, to generalize responses.

## Perspectives

Molluscs are the taxon with the highest number of extinctions; 42% of the 693 recorded extinctions of animal species since the year 1500 were molluscs, and 99% of them were terrestrial and freshwater species (Lydeard *et al*., 2004). As a result of a general bias in both public and scientific interest for vertebrate extinctions, the conservation status of gastropod species is poorly known and biodiversity management generally not intended to promote their maintenance (Régnier *et al*., 2008, 2015).

To understand how a species will be affected by climate change, we need to know responses at different organizational levels: species-specific physiological and behavioural processes, population responses and distribution of species in the climatic heterogeneous landscape. In most endangered gastropods, studies are scarce and sensitivity traits (physiological and behavioural) and their evolvability unknown. Without this knowledge, we are unable to assess species’ status that will be the basis for species’ recovery strategies, integrated in conservation strategies of ecosystems.

Experimental manipulation of environmental conditions in the field (temperature and/or rainfall; Sternberg, 2000) or in climate chambers (temperature and/or CO_2_; Bezemer and Knight, 2001) has confirmed the difficulty in drawing a general picture of organisms’ responses. In general, physiological studies, involving metabolomic fingerprinting, survival or performance analysis in different climatic conditions (methods used in studies of Table 1; for more, see Madliger and Love, 2015), are highly invasive, making them unsuitable for endangered and protected species. As numerous physiological processes are phylogenetically constrained, an ideal situation would be to approximate the responses of endangered species by those of a common, phylogenetically close species. However, our example of cold tolerance in three Helicidae species showed that they differ in life-history strategy, behavioural responses and physiological processes. Therefore, species-specific studies at the population level and the development of non-invasive methods (e.g. heart rate to measure thermal performance; Marshall and McQuaid, 2011; Han *et al*., 2013) are required to understand the physiological responses to climate change of rare and endangered species.

Habitat alteration drives species to migrate to suitable habitat. Given that most of the critically endangered species are on islands (Fig. 1 and, e.g. COSEWIC, 2014, in press), habitat is limited. Moreover, human-driven habitat loss and fragmentation through intensified urbanization, agriculture and forestry, dramatically increases the impact of climate change at the population level. Especially for endangered species, living in restricted habitat (such as protected areas or habitat remnants), dispersal or migration being largely negated by habitat loss and fragmentation, studies on the impact of habitat alteration are still scarce, but greatly needed.

Recently, several authors pointed to the necessity of integrating physiological and behavioural traits in an interacting environment to avoid false predictions (Helmuth, 2009; Kearney *et al*., 2009; Chapperon and Seuront, 2011; Huey *et al*., 2012; Twomey *et al*., 2012). Historical impacts of climate change and anthropogenic pressure on population size or on habitat structure combined with density-dependant population maintenance processes, such as reproduction and dispersal, should be considered to develop dynamic conservation models in changing landscape. Providing a thermally heterogeneous landscape may be a key component of conservation of terrestrial species under climate change. However, the complexity of the thermal landscape as perceived by an organism is difficult to infer from average temperatures recorded by weather stations, so many studies are simplified to take only one landscape factor into account, e.g. topography (Sears *et al*., 2011). Anthropogenic activity influences these landscape parameters, but is often largely ignored in large-scale models. The link between habitat choice and physiology is poorly known for all but a few species (Angilletta, 2009). Genetic diversity, phenotypic plasticity and evolvability of traits seem to be essential elements to a complete understanding of how a species will face climate change (Marshall *et al*., 2010; Donnelly *et al*., 2012; Huey *et al*., 2012; Merilä and Hendry, 2014; Schilthuizen and Kellermann, 2014). Integrative approaches should consider spatiotemporal heterogeneity of climate at local scales over a species’ range and the capacity of species to respond to climate variations through plasticity of traits or adaptation.

Understanding the spatiotemporal effect of climate change on gastropod species would require a precise knowledge of specific physiological processes, behavioural responses and their plasticity in combination with genetic adaptation at different organizational scales, from individual to species. This objective seems reachable for some well-studied model species, such as the brown garden snail *C. aspersum* or the grove snail *C. nemoralis*, with a panel of studies conducted worldwide, from phylogeographic history (e.g. Davison and Clarke, 2000; Guiller *et al*., 2012) to physiological (see Table 1) and behavioural traits (e.g. Jaremovic and Rollo, 1979; Dahirel *et al*., 2014). Extrapolation to other species, particularly to endangered ones, remains limited, and a minimal knowledge of basic physiological processes in species at risk is urgently needed to determine adequate conservation strategies.

In parallel, as most terrestrial gastropods are part of a diverse mulch and litter community that is essential in all ecosystems to ensure soil fertility (Swift *et al*., 1979), it is necessary to develop a functional approach to understand the effect of climate change in the soil system. The need for the preservation of soil functions, via the conservation of species in the litter community and the ecosystems they occupy, is still too often underestimated.
